# The Impact of Javanica Oil Emulsion Injection on Chemotherapy Efficacy and Cellular Immune Indicators in Patients with Advanced NSCLC: A Systematic Review and Meta-Analysis

**DOI:** 10.1155/2019/7560269

**Published:** 2019-10-22

**Authors:** Huilin Xu, Zhucheng Yin, Anbing He, Dedong Cao

**Affiliations:** ^1^Department of Oncology, The Fifth Hospital of Wuhan, Wuhan 430000, China; ^2^Department of Oncology, Hubei Cancer Hospital, Tongji Medical College, Huazhong University of Science and Technology, Wuhan 430000, China; ^3^Department of Oncology, RenMin Hospital of Wuhan University, Wuhan 430000, China

## Abstract

**Background:**

This meta-analysis aimed to evaluate the efficacy and safety of Javanica oil emulsion injection (JOI) combined with chemotherapy versus chemotherapy in patients with advanced non-small-cell lung cancer (NSCLC).

**Methods:**

Electronic databases including EMBASE, PUBMED, the Cochrane library, and Chinese Biological Medical disc (CBM) were searched until May 2018. The clinical trials reporting efficacy and immune function of JOI combined with chemotherapy versus chemotherapy in advanced NSCLC were included according to the inclusion and exclusion criteria. Stata 11 and RevMan 5.3 were used for meta-analysis.

**Results:**

Twenty-four studies involving 2089 cases were included. The results of the meta-analysis showed that there were significant differences in objective response rate (risk ratio (RR) = 1.17; 95% confidence interval (CI): 1.05–1.29; *P* < 0.05), improvement in Karnofsky Performance Status (standard mean difference (SMD) = 1.59; 95% CI: 1.41–1.77; *P* < 0.01), incidence of adverse events (RR = 0.78; 95% CI: 0.7–0.87; *P* < 0.05), percentage changes of CD_3_^+^ cells (SMD = 2.0; 95% CI: 1.49–2.50; *P* < 0.01), CD_4_^+^ cells (SMD = 1.55; 95% CI, 1.2–1.9; *P* < 0.01), natural killer cells (SMD = 1.98; 95% CI: 1.15–2.82; *P* < 0.01), but not CD_8_^+^ (SMD = −1.44; 95% CI: −4.53–1.65; *P*=0.36), and value of CD_4_^+^/CD_8_^+^ (SMD = 0.32; 95% CI: 0.28–0.36; *P* < 0.01) between the JOI combination group and control group. Funnel plot and Begg's and Egger's analysis indicated that there was no significant publication bias (*P* > 0.05).

**Conclusions:**

JOI may be effective to improve the efficacy of chemotherapy in advanced NSCLC patients, accompanied with better levels of immune cells.

## 1. Introduction

Non-small-cell lung cancer (NSCLC) is the most common type of lung cancer, accounting for 80%–85% of lung cancer [1]. It has become one of the most lethal tumors worldwide [1]. Most patients are diagnosed with advanced disease at the time of diagnosis and missed the chance of radical surgery [2]. These patients usually receive treatments including targeted therapy, chemotherapy, radiotherapy, and other palliative care [2, 3]. Although new treatment options can significantly improve the prognosis of these patients, chemotherapy still plays an important role in the treatment of advanced disease [4].

The CD_3_^+^, CD_4_^+^, and CD_8_^+^ cell subsets belong to T lymphocytes and play important roles in antitumor immunity [5, 6]. The CD_8_^+^ cell subpopulation, also known as cytotoxic T cells, participates in the regulation of the body's immune balance [6, 7]. CD_4_^+^/CD_8_^+^ ratio is an important indicator reflecting the body's immune status and cellular immune function [8]. Patients with advanced cancer show reduced immune function, exhibiting imbalance of T lymphocytes percentage, function, and decreased natural killer cell activity [9, 10]. Chemotherapy agents could have a negative impact on the immune function, so that the immune function of tumor patients may be further impaired by chemotherapy, therefore ultimately affecting the therapeutic effect [11].

Javanica oil emulsion injection (JOI) is a traditional Chinese medicine preparation that proves to kill tumor cells while protecting the body's immune function [12–14]. The JOI is an oil-in-water emulsion made by emulsifying fatty oil extracted from mature seeds of *Brucea javanica* [12–14]. The main anticancer active ingredients are oleic acid and linoleic acid [12–14]. Preclinical studies have shown that the antitumor mechanisms of JOI are designed through inhibiting the activity of topoisomerase and the synthesis of DNA in tumor cells [13–16]. At the same time, the tiny oil particles of *Brucea javanica* oil have specific affinity with the tumor cells and can adhere to the tumor cells for a long time, which is beneficial for the penetration of antitumor components into the tumor cells. This may result in reducing the damage to the normal tissue cells and the risk of adverse events of chemotherapy [14, 17–19]. JOI can also activate the body's immune system and restore immunity [20, 21]. The study conducted by He et al. showed that an improved efficacy was observed in NSCLC patients treated with JOI combined with chemotherapy, associated with reduced serum levels of interleukins, tumor necrosis factor, and other inflammatory factors [20]. Although previous meta-analysis [22] evaluated the effect of JOI plus chemotherapy, the attention was focused on efficacy and safety but not immune function. Therefore, updated evidence evaluating the impact of *Brucea javanica* oil injection on efficacy and immunity in patients with advanced NSCLC is needed.

In this study, we systematically searched several databases, extracted relevant data, and analyzed the impact of JOI combined with chemotherapy versus chemotherapy on efficacy and immune function in advanced NSCLC patients.

## 2. Methods

### 2.1. Search Strategy

This study was performed based on the Preferred Reporting Items for Systematic Reviews and Meta-Analyses (PRISMA) guidelines [23]. The search strategy was developed according to the Cochrane Collaboration Handbook 5.1. Electronic databases including EMBASE, PUBMED, the conference proceedings of the American Society of Clinical Oncology (ASCO), the Cochrane library, and Chinese Biological Medical disc (CBM) were searched until May 2018 to identify clinical trials and/or randomized controlled trials (RCTs) of JOI combined with chemotherapy versus chemotherapy for advanced NSCLC. The search terms were Javanica oil emulsion injection, Javanica injection, Brucea oil injection, non-small-cell lung cancer, NSCLC, immunity, and immune function. The “similar articles” function in PUBMED was used to further identify potential eligible studies. No language limitation was applied in this study.

### 2.2. Inclusion and Exclusion Criteria

(1) Patients: advanced NSCLC diagnosed by sufficient evidence, such as cytology and/or pathology and imaging exams. The advanced disease was defined as stage III B and/or IV NSCLC; (2) type of study: RCTs and/or retrospective studies; (3) intervention: JOI plus platinum-based chemotherapy versus platinum-based chemotherapy; (4) outcomes: primary endpoints were efficacy and survival rates. Secondary endpoints were cellular immune function indicators such as percentages of total T lymphocytes (CD_3_^+^), helper T lymphocytes (CD_4_^+^), cytotoxic T lymphocytes (CD_8_^+^), ratio of CD_4_^+^/CD_8_^+^ and natural killer cells (NK), quality of life, and incidence of adverse events related to chemotherapy. Animal studies, case reports, reviews, clinical experience, and duplicated literature were excluded.

### 2.3. Study Selection

The abstracts and topics of the retrieved articles were screened by two reviewers, independently. The remaining studies were further reviewed to determine if they met the above inclusion criteria. If there was disagreement with regard to the results of the study selection after cross-checking, it was discussed and resolved by a third reviewer.

### 2.4. Data Extraction and Quality Assessment

The extracted data were: (1) general information, such as study title, name of the first author, time of publication, and source of the literature; (2) baseline data including sex, age, diagnostic criteria, number of participants, description of the reasons for withdrawal, or follow-up visits lost; (3) design and implementation data including study type, duration of follow-up, interventions, and measurement units; (4) Outcome indicators, such as changes of cellular immune function indicators (percentages of CD_3_^+^, CD_4_^+^, CD_8_^+^, and NK cells), overall response rate (ORR), disease control rate (DCR), quality of life (QoL), Karnofsky Performance Scores (KPS), survival rates, and incidence of adverse events. The author was contacted via email if it was necessary to access sufficient data.

The methodological quality of included RCTs was assessed according to the RCT quality assessment criteria reported in the Cochrane Reviewer Handbook 5.1.4. [24] There were six aspects, such as random assignment, blind grouping, blind implementation, incomplete data, selective report, and other potential bias. The bias risk of each eligible study was assessed by two reviewers, independently. In case of inconsistent opinions, the disagreement was discussed or negotiated or consulted with a third investigator. The Newcastle–Ottawa scale (NOS) was introduced to evaluate the quality of the retrospective studies [25]. Three major aspects including selection, comparability of the cohort, and evaluation of the results are mainly focused for assessment. According to the NOS criteria, the selection, the comparability, and the results assessment can be assigned with a maximum of 4 stars, 2 stars, and 3 stars, respectively. Study with six or more stars was considered as good quality.

### 2.5. Statistical Analysis

The software Stata 11 and RevMan 5.3 were used to conduct the pooled analysis. The statistical methods were similar as previously described [26]. Briefly, the pooled risk ratio (RR) along with its 95% confidence interval (CI) was calculated to present dichotomous data. For continuous data, the weighted mean differences (WMD) and its associated 95% CI were calculated if the unit of the data was consistent, otherwise the SMD was used. We used *Z*-test analysis and *I*^2^ test to assess the overall heterogeneity across the included studies. As indicated by the value of *I*^2^%, 0%–40% indicates low risk of heterogeneity, 30%–60% indicates moderate risk of heterogeneity, 50%–90% indicates significant risk of heterogeneity, and 75%–100% indicates a greater significant risk of heterogeneity [24]. The random effect model was applied if there was significant heterogeneity between the included studies, or the fixed effect model was used. The main endpoints of this study were objective response rates (ORR), survival rate, changes of percentages of CD_3_^+^, CD_4_^+^, CD_8_^+^, ratio of CD_4_^+^/CD_8_^+^, and NK from baseline to after treatment [24], the QoL improvement, and incidences of adverse events. To detect publication bias with regard to the immune function indicators and response rate, the funnel plot and Egger's test were used. *P* < 0.05 was considered as there was a significant difference.

## 3. Results

### 3.1. Search Results

**Figure 1:**
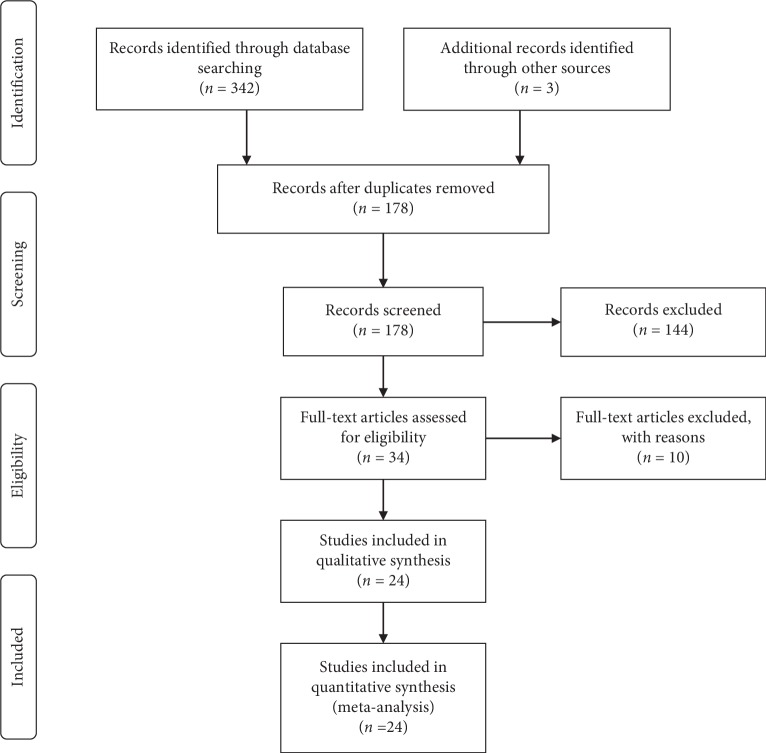
Flow diagram of searching for eligible studies.

Through preliminary search, a total of 345 related articles were obtained. After removing duplication and reading of the title and the abstract, 167 studies were discarded, and the remaining 178 articles were included for further review. After reading the full text, 144 articles were further excluded, and 24 studies [18, 20, 21, 27–47] were finally considered as eligible studies. A total of 2089 NSCLC patients were included, with 1060 in the JOI plus chemotherapy group and 1029 cases in the chemotherapy group. The literature screening process and study selection results are shown in Figure 1.

### 3.2. Baseline Characteristics of Identified Studies

The baseline characteristics of included studies are listed in Table 1. 92% of these studies were RCTs, and only 2 were retrospective studies. The publication time ranged from 2006 to 2017. These patients were diagnosed with advanced NSCLC. The duration of the JOI treatment ranged from 10 days to 50 days. The interventions of JOI plus gemcitabine plus platinum (GP) regimen versus GP regimen were used to treat patients in twelve studies, four used JOI plus Taxotere plus platinum (TP) regimen versus TP regimen, JOI plus vinorelbine plus platinum (NP) regimen versus NP regimen in six studies, and two studies used JOI plus platinum-based regimen versus platinum-based regimen to treat NSCLC. 21 of the studies reported outcomes of tumor response, all showed changes of immune function before and after treatments, 13 studies showed incidences of adverse events, and fourteen reported improvement in quality of life. The overall quality of the included studies is presented in Table 2.

### 3.3. Efficacy of JOI Combined with Chemotherapy

**Figure 2:**
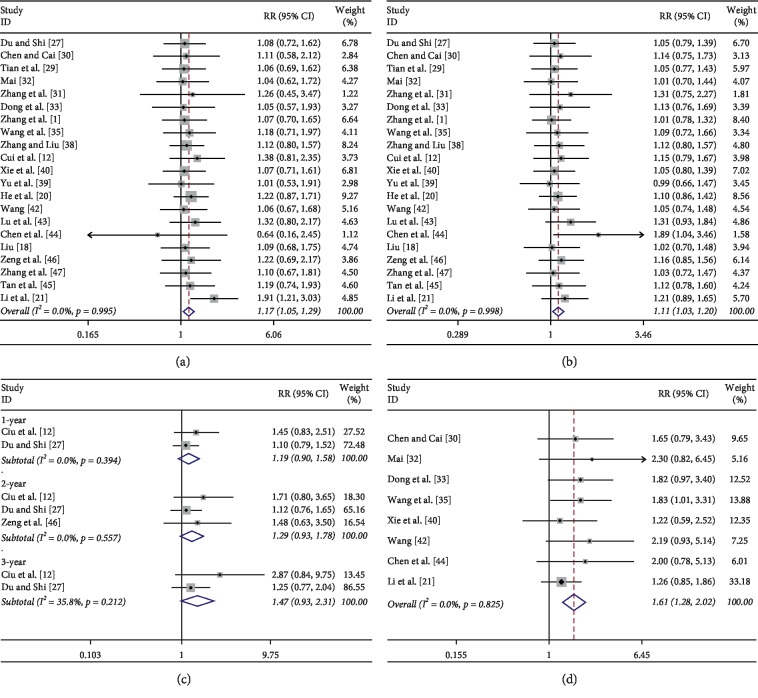
Pooled results of ORR, DCR, and survival rates and improvement in quality of life of JOI combined with chemotherapy versus chemotherapy for advanced NSCLC patients. (a) ORR; (b) DCR; (c) Survival rates; (d) improvement of quality of life.

A total of 21 studies [18, 20, 21, 27, 29–33, 35–40, 42–47] reported the overall response rate in patients with advanced NSCLC treated with JOI combined with chemotherapy versus chemotherapy alone. The result of meta-analysis showed that it was homogenous when pooling disease control rates data together (heterogeneity test *P*=0.995, *I*^2^ = 0%), so the fixed effect model was used. As shown in Figure 2(a), the total number of treatment-responded cases in the JOI-combined chemotherapy group was 534 (53.9%), while 413 cases (43%) achieved treatment response in the chemotherapy alone group. The disease control rates [18, 20, 21, 27, 29–33, 35–40, 42–47] in the two groups were significantly different, and the combined effects were RR = 1.11 (95% CI: 1.03–1.20; *P* < 0.05), indicating that the rate of disease control in the JOI-combined chemotherapy group was significantly better than that in the chemotherapy alone group (Figure 2(b)). The results of disease control rates in the three subgroups were as follows: JOI + TP vs TP alone (RR = 1.08; 95% CI: 0.91–1.29; *P* > 0.05), JOI + NP vs NP alone (RR = 1.09; 95% CI: 0.91–1.31; *P* > 0.05), JOI + GP vs GP alone (RR = 1.11; 95% CI: 1.01–1.22; *P* < 0.05). The results of the sensitivity analysis suggested that neither the RR nor the 95% CI would change significantly if excluding either one of the included studies.

Only three RCTs [27, 37, 46] reported survival rate in terms of 1 year, 2 year, and 3 year. The combined results illustrated by Figure 2(c) showed that there were no significant differences in survival rates at different time points between JOI and chemotherapy versus chemotherapy (all *P* > 0.05).

Another concern was the impact on the quality of life. We extracted data about number of patients who had improved quality of life, and the combined estimate from eight RCTs [21, 30, 32, 33, 35, 40, 42, 44] showed that adding JOI to chemotherapy could significantly improve the overall quality of life (RR = 1.61; 95% CI: 1.28–2.02; *P* < 0.01, Figure 2(d)). Due to significant heterogeneity among the included studies, the changes of KPS were similar between different groups (data not shown).

### 3.4. Changes of Cellular Immunity

**Figure 3:**
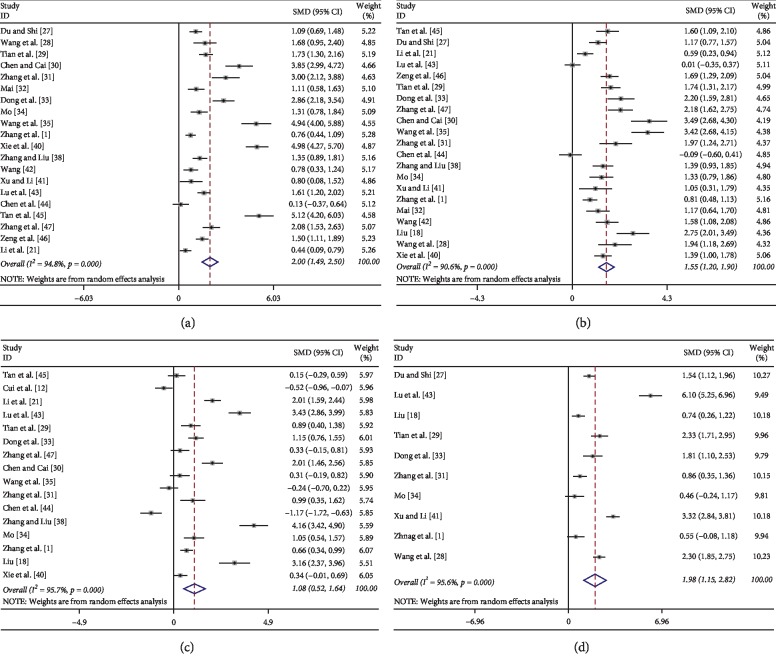
Meta-analysis of changes of immune cells percentages when JOI combined with chemotherapy in patients with advanced NSCLC. (a) percentage of CD_3_^+^ cells; (b) percentage of CD_4_^+^ cells; (c) ratio of CD_4_^+^/CD_8_^+^; (d) percentage of NK cells.

Twenty studies [21, 27–36, 38, 40–47] provided changes of percentage of CD_3_^+^ cells after treatment. The random effect model applied as a statistical significant heterogeneity was found (*I*^2^% = 94.8%, *P* < 0.01). The results showed that the SMD was 2.00 (95% CI: 1.49–2.5; *P* < 0.01), indicating that adding JOI to chemotherapy was associated with increased percentage of total lymphocytes in advanced NSCLC (Figure 3(a)).

Twenty-one articles [21, 27–29, 31–47] exhibited changes of percentages of helper T lymphocytes after treatment. These data were not homogeneous (*I*^2^% >50%), so the random effect model was applied. As shown in Figure 3(b), patients in JOI plus chemotherapy had an advantage of increased percentage of helper T lymphocytes compared to that of the chemotherapy group (SMD = 1.55; 95% CI: 1.20–1.90; *P* < 0.01).

There were 23 trials [18, 21, 27–47] which reported improvement of percentages of cytotoxic T lymphocytes. Significant heterogeneity was found among the included studies (*I*^2^% >50%), and the random effect model was used. The results illustrated that the changes of CD_8_^+^ cells were similar between the JOI combination group and chemotherapy alone group (SMD = 0.07; 95% CI: −0.51–0.65; *P* > 0.05) (Supplemental [Supplementary-material supplementary-material-1]).

The ratio of CD_4_^+^/CD_8_^+^ was reported in seventeen studies [18, 21, 28–31, 33, 34, 36–40, 43–45, 47]. The random effect model was applied as we detected a statistically significant heterogeneity among these studies (*I*^2^% = 95.7%). As shown in Figure 3(c), there was no significant difference in changes of CD_4_^+^/CD_8_^+^ ratio between the JOI plus chemotherapy group versus chemotherapy group (SMD = 1.08; 95% CI: 0.52–1.64; *P* > 0.05).

Ten articles [18, 27, 28, 31, 33, 34, 36, 40, 41, 43] reported changes of levels of NK cells after treatment. The *I*^2^ test found that there was no homogeneity among the included studies (*I*^2^% >50%), so the random effect model was used. As shown in Figure 3(d), adding JOI to chemotherapy was associated with a significant increase in the percentage of NK cells compared with the chemotherapy group (SMD = 1.98; 95% CI: 1.15–2.82; *P* < 0.01).

### 3.5. Detoxication of JOI Combined with Chemotherapy

**Figure 4:**
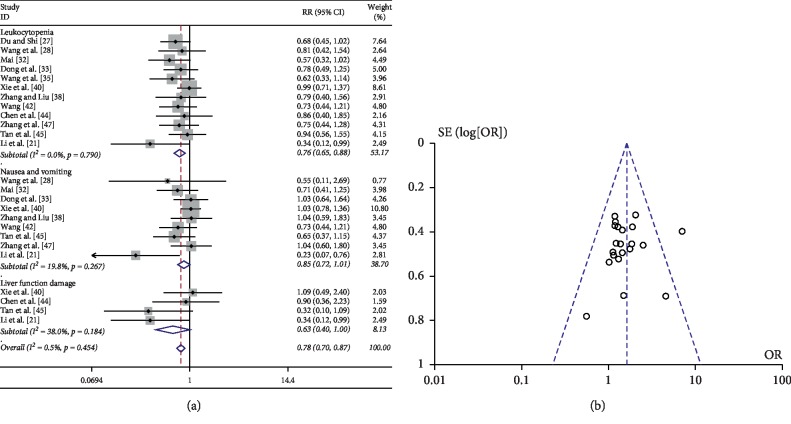
(a) Meta-analysis of JOI on adverse events when combined with chemotherapy in patients with advanced NSCLC. (b) Funnel plot used to assess publication bias.

There were 12 [21, 27, 28, 32, 33, 35, 38, 40, 42, 44, 45, 47], 9 [21, 28, 32, 33, 38, 40, 42, 45, 47], and 4 [21, 40, 44, 45] studies which reported the incidence of leukocytopenia, nausea, and vomiting, and liver function damage in patients with advanced NSCLC treated with JOI combined with chemotherapy versus chemotherapy alone, respectively. The heterogeneity test results showed that it was homogenous among these studies (*P* > 0.1). Therefore, the combined effects were calculated using relative risk based on the fixed effect model. As shown in Figure 4(a), there were differences in the incidences of leukocytopenia and liver function injury between the two groups. The combined effect of RR was 0.76 (95% CI: 0.65, 0.88) for leukocytopenia and 0.63 (95% CI: 0.40, 1.00) for liver function damage, suggesting that the incidences of chemotherapy-caused adverse events in the JOI-combined chemotherapy group were lower to those of the chemotherapy alone group.

### 3.6. Publication Bias

The funnel plot analysis of the included studies was conducted using data of the total response rate. The symmetry of the funnel graph is good (Figure 4(b)), suggesting that the results are less likely to be affected by publication bias (Begg's test : *P*=0.928; Egger's test: *P*=0.495).

## 4. Discussion

In this study, clinical studies of JOI combined with chemotherapy versus chemotherapy alone in the treatment of advanced NSCLC were included to evaluate the efficacy and safety after treatments. The meta-analysis confirmed that the JOI-combined chemotherapy was significantly better than the chemotherapy alone in terms of overall response rate, disease control rate, and improvement in quality of life, along with better changes of cellular immune function indicators, such as percentages of CD_3_^+^, CD_4_^+^, and NK cells. With regard to safety, the incidences of myleosuppression in JOI combined with chemotherapy were significantly lower than that of the chemotherapy group. JOI-combined chemotherapy group and chemotherapy alone group shared similar incidences in gastrointestinal adverse events and liver function damage.

Previously, three meta-analyses [19, 22, 48] focusing on the efficacy and safety of JOI in treating lung cancer were reported. In 2012, Wang et al. [22] performed an academic search and used meta-analysis method to study the efficacy and safety of JOI combining platinum-contained first-line chemotherapy in treating NSCLC. By including 22 RCTs involving 1512 patients, they found that the chance to gain a better disease control in the JOI and chemotherapy combination group was 1.31 times when compared with chemotherapy alone. These patients also benefited from 1.78 times of improved quality of life and 0.37 times of myelosuppression. Another study [19] by Nie et al. was also published in 2012. They evaluated the effectiveness and safety of JOI plus chemoradiotherapy to alleviate suffer of lung cancer patients. They reported that JOI may improve immune function in lung cancer. However, their results were obtained based on high risk of bias. The intervention and disease type of included studies varied. These studies not only included advanced stage disease but also early or moderate stages, and even small-cell lung cancer, which may bring high risk of bias and heterogeneity and make the findings less reliable. Few years later, Xu and other researchers [48] evaluated the efficacy of JOI in advanced NSCLC during chemotherapy. They included 21 studies involving 2234 cases. Their results showed a lower RR for response and a higher RR for myelosuppression than those of Wang's. Their conclusion was that JOI could enhance efficacy and improve quality of life and common adverse events during chemotherapy for advanced NSCLC. They did not evaluate the changes of immune function variables after JOI combination therapy. In our study, we only included advanced NSCLC patients receiving JOI and chemotherapy versus chemotherapy alone, with adequate data for both efficacy and changes of percentage of immune cells, ensuring a low risk of heterogeneity and reliable pooled estimates.

As previously reported, CD_3_^+^ T lymphocytes accounts for 10% to 20% of the first trimester human decidual leukocyte population. Among these CD_3_^+^ lymphocytes, 40% to 75% of them are CD_8_^+^ cytotoxic T lymphocytes (CTLs), and 30% to 45% are CD_4_^+^ helper T cells (Th) [49]. T lymphocytes play important roles in fighting against various diseases and can affect the antitumor effects of other immune cells [5, 6, 49]. In this study, we observed that the overall percentages of CD_3_^+^ and CD_4_^+^ cells were decreased in either JOI combination therapy group or chemotherapy alone group. We found that the percentages of CD_3_^+^ and CD_4_^+^ cells in the combination group were 2% and 1.55% more than those in the chemotherapy group, respectively. This indicates that JOI may protect these cells from chemotherapy. The CD_8_^+^ cell subpopulation is known as cytotoxic T cell, with the function of regulating the body's immune balance [6, 7]. We did not find a significant change in levels of CD_8_^+^ cells when comparing different treatment groups, as well as the CD_4_^+^/CD_8_^+^ ratio, which is an important indicator reflecting the body's immune status and cellular immune function [8]. However, we observed that the pooled percentage of NK cells in the JOI group was 1.98% more than that of the chemotherapy group, which may partially explain the better ORR and DCR in the combination group. These findings may reflect the immune function enhancement by JOI indirectly, and more efforts are needed to verify the actual impact on immune functions and its underlying mechanisms.

In this meta-analysis, the *I*^2^% was found to be relatively high in some evaluations, indicating that there was obvious heterogeneity among these analyses. The main reason may be as following: the types of some research were retrospective studies, though there were also randomized controlled trials; the purpose and size of each trial varied; the patient's baseline characteristics (clinical stage, average age, gender, race, region, etc.), treatment, severity of the disease, dose of medication, and the duration of treatment also varied in some extent. Although the random effect model was used in the analysis, and the influence of heterogeneity was eliminated to some extent, it could not completely avoidable. Therefore, large sample, multi-center studies are needed to confirm the findings.

The results of the quality assessment of the research literature showed that the included studies were of moderate quality and only few of them showed specific randomization method was used during the process of randomization. The rest of the studies did not show their specific stochastic and allocation concealment methods. Although our study included as many as 24 articles, the number of patients in each trial was relatively small, and the existence of publication bias may be inevitable. This may be related to the published literature had positive findings. The lost ones could be grey literature, such as unpublished literature, unpublished results due to negative results, special reports, and so on. These factors were bound to affect the results of our meta-analysis and may even amplify interventions. With regards to the materials, methods, and timing of analysis about immune function, we found that the materials used for analysis were peripheral blood samples, the methods used to detect the percentages of different immune cells were flow cytometry, and the methods used to measure the concentrations of cytokines were enzyme-linked immunosorbent assay. The timings to test the samples were at baseline and posttreatment. As all the samples were peripheral blood, and the measurement methods were the same type, we consider there was low risk of selection and measurement bias with regards to the samples and methods. Although all the included studies reported the test timings were before and after treatments, the exact time to collect the samples were not mentioned, increasing the risk of selective reporting. In view of the above defects and problems, it was suggested that the results of this study should be applied cautiously.

## 5. Conclusions

The results of this study showed that JOI combined with chemotherapy was effective in treatment of advanced NSCLC patients, and accompanied with better percentages of immune cells. However, the overall quality of our evidence is moderate. Further research is needed to verify the efficacy of this therapy.

## Figures and Tables

**Table 1:** Baseline characteristics of included studies.

Studies	Year	Number of patients		Age		Sex		Intervention		Sample	Test timing	Followed-up	Outcomes
		T	C	T	C	Male	Female	T	C				
Min Du	2006	56	57	27–72	27–72	76	37	JOI 30 mL, iv, qd, 14d + NP	NP	Peripheral blood	Baseline and posttreatment	3 years	IF, 1, 2, 3-year SR, RR, SF-36, AE
Haiyong Wang	2006	20	20	49–81	45–75	29	11	JOI 50 mL, iv, qd, 14d + NP	NP	Peripheral blood	Baseline and posttreatment	3 months	IF, AE
Huaqin Tian	2007	58	57	43–74	42–78	79	36	JOI 40 mL, iv, qd, 14d + GP	GP	Peripheral blood	Baseline and posttreatment	3 months	IF, KPS, RR
Xi Chen	2007	30	30	35–72	37–73	41	19	JOI 30 mL, iv, qd, 10d + NP	NP	Peripheral blood	Baseline and posttreatment	2 months	IF, KPS, RR, AE
Sufang Zhang	2008	22	21	70–83	70–83	29	14	JOI 40 mL, iv, qd, 14d + GP	GP	Peripheral blood	Baseline and posttreatment	3 months	IF, RR
Zefeng Mai	2008	35	30	30–72	31–72	40	25	JOI 40 mL, iv, qd, 20d + TP	TP	Peripheral blood	Baseline and posttreatment	2 months	IF, KPS, RR, AE, weight
Xilin Dong	2009	34	34	60–79	62–78	42	26	JOI 30 mL, iv, qd, 14d + NP	NP	Peripheral blood	Baseline and posttreatment	3 months	IF, KPS, RR
Shaoxiong Mo	2010	33	33	55.6 ± 4.5	54.8 ± 5.3	42	24	JOI 30 mL, iv, qd, 14d + GP	GP	Peripheral blood	Baseline and posttreatment	3 months	IF
Ken Wang	2011	36	36	32–75	32–75	42	30	JOI 50 mL, iv, qd, 30d + NP	NP	Peripheral blood	Baseline and posttreatment	2 months	IF, KPS, RR, AE
Yu Zhang	2011	82	74	23–74	28–73	103	53	JOI 30 mL, iv, qd, 14d + GP	GP	Peripheral blood	Baseline and posttreatment	2 months	IF, RR
Yi Cui	2014	40	40	NA	NA	45	35	JOI 30 mL, iv, qd, 14d + TP	TP	Peripheral blood	Baseline and posttreatment	3 years	IF, 1, 2, 3-year SR, RR, weight, CEA, CA125
Yinzi Zhang	2014	45	45	67.1 ± 5.9	66.7 ± 5.3	61	29	JOI 40 mL, iv, qd, 14d + TP	TP	Peripheral blood	Baseline and posttreatment	3 months	IF, KPS, RR, AE
Lili Yu	2014	30	26	33–77	33–77	44	12	JOI 30 mL, iv, qd, 10d + platinum-based CT	Platinum-based CT	Peripheral blood	Baseline and posttreatment	2 months	IF, KPS, RR, AE
Weibo Xie	2014	63	63	67 ± 15.4	61.1 ± 10.9	83	43	JOI 30 mL, iv, qd, 30d + GP	GP	Peripheral blood	Baseline and posttreatment	4 months	IF, KPS, RR, AE
Daojing Xu	2015	16	16	61.8 ± 13.1	62.4 ± 13.7	19	13	JOI 50 mL, iv, qd, 14d + NP	NP	Peripheral blood	Baseline and posttreatment	3 months	IF
Li Wang	2015	40	40	50 ± 2.34	50 ± 2.34	58	22	JOI 30 mL, iv, qd, 10d + TP	TP	Peripheral blood	Baseline and posttreatment	1 month	IF, KPS, RR, AE, weight
Min He	2015	80	80	63.72 ± 7.5	64.18 ± 8.19	119	41	JOI 50 mL, iv, qd, 10d + GP	GP	Peripheral blood	Baseline and posttreatment	3 years	IF, RR, SR
Yishan Lu	2016	60	60	64.8 ± 1.7	66.0 ± 3.7	74	46	JOI 30 mL, iv, qd, 14d + GP	GP	Peripheral blood	Baseline and posttreatment	4 months	IF, RR
Yan Chen	2016	30	30	59.6 ± 8.7	59.8 ± 10.5	39	21	JOI 30 mL, iv, qd, 10d + platinum-based CT	Platinum-based CT	Peripheral blood	Baseline and posttreatment	3 months	IF, KPS, RR, AE
Baoli Tan	2017	40	40	52.2 ± 8.5	52.8 ± 8.1	55	25	JOI 20 mL, iv, qd, 10d + GP	GP	Peripheral blood	Baseline and posttreatment	6 months	IF, VEGF, AE, KPS, 0.5-year SR, RR
Yong Zeng	2017	71	60	59.4 ± 7.9	60.2 ± 9.2	78	53	JOI 20 mL, iv, qd, 14d + GP	GP	Peripheral blood	Baseline and post-treatment	2 years	IF, AE, RR, 2-year SR
Ying Liu	2017	35	35	58.6 ± 3.5	58.9 ± 3.2	45	25	JOI 20 mL, iv, qd, 14d + GP	GP	Peripheral blood	Baseline and posttreatment	2 months	IF, KPS, RR
Bo Zhang	2017	39	39	67 ± 11	68 ± 12	54	24	JOI 40 mL, iv, qd, 14d + GP	GP	Peripheral blood	Baseline and posttreatment	2 months	IF, KPS, RR
Hui li	2018	65	63	57 ± 4	56 ± 4	68	60	JOI 30 mL, iv, qd, 14d + GP	GP	Peripheral blood	Baseline and posttreatment	3 months	IF, KPS, RR, AE

T, treatment; C, control; RR, response rate; IF, immune function; JOI, Javanica oil injection; TP, Taxotere + Platinum; NP, Vinorelbine + Platinum; GP, Gemcitabine + Platinum; CT, chemotherapy; KPS, Karnofsky Performance Status; VEGF, vascular endothelial growth factor; SR, survival rate; AE, adverse events; iv, Intravenous therapy; qd, one a day; d, day.

**Table 2:** Quality assessment of included studies.

Studies	Year	Randomization	Allocation	Blinding	Integrity of results	Selective reporting	Lost	Other bias	Randomization method
Min Du	2006	Y	N	N	N	N	N	NR	Y
Haiyong Wang	2006	Y	N	N	N	N	N	NR	N
Huaqin Tian	2007	Y	N	N	N	N	N	NR	Y
Xi Chen	2007	Y	N	N	N	N	N	NR	N
Sufang Zhang	2008	Y	N	N	N	N	N	NR	N
Zefeng Mai	2008	Y	N	N	N	N	N	NR	N
Xilin Dong	2009	Y	N	N	N	N	N	NR	Y
Shaoxiong Mo	2010	Y	N	N	N	N	N	NR	N
Ken Wang	2011	Y	N	N	N	N	N	NR	N
Yu Zhang	2011	Y	N	N	N	N	N	NR	N
Yi Cui	2014	Y	N	N	N	N	N	NR	N
Yinzi Zhang	2014	Y	N	N	N	N	N	NR	N
Lili Yu	2014	Y	N	N	N	N	N	NR	N
Weibo Xie	2014	Y	N	N	N	N	N	NR	N
Daojing Xu	2015	N	N	N	N	N	N	NR	N
Li Wang	2015	Y	N	N	N	N	N	NR	N
Min He	2015	Y	N	N	N	N	N	NR	Y
Yishan Lu	2016	N	N	N	N	N	N	NR	N
Yan Chen	2016	Y	N	N	N	N	N	NR	Y
Baoli Tan	2017	Y	N	N	N	N	NR	NR	Y
Yong Zeng	2017	Y	N	N	N	N	N	NR	Y
Ying Liu	2017	Y	N	N	N	N	N	NR	N
Bo Zhang	2017	Y	N	N	N	N	N	NR	N
Hui li	2018	Y	N	N	N	N	N	NR	N

Y, yes; NA, not available; NR, not reported; N, no.

## Abbreviations

NSCLC: Non-small-cell lung cancer
JOI: Javanica oil emulsion injection
CBM: Chinese biological medical disc
RR: Risk ratio
CI: Confidence interval
SMD: Standard mean difference
PRISMA: Preferred Reporting Items for Systematic Reviews and Meta-Analyses guidelines
ASCO: American Society of Clinical Oncology
RCTs: Randomized controlled trials
NK: Natural killer cells
QOL: Quality of life
KPS: Karnofsky Performance Status
WMD: Weighted mean differences
ORR: Objective response rate
GP: Gemcitabine plus platinum
TP: Taxotere plus platinum
NP: Vinorelbine plus platinum.

## Supplementary Materials

Supplementary Materials Supplemental Figure 1: meta-analysis of change of CD_8_^+^ percentage when JOI combined with chemotherapy versus chemotherapy in patients with advanced NSCLC. Supplemental Table 1: Preferred Reporting Items for Systematic Reviews and Meta-Analyses checklist.
